# Folic Acid-Targeted Paclitaxel-Polymer Conjugates Exert Selective Cytotoxicity and Modulate Invasiveness of Colon Cancer Cells

**DOI:** 10.3390/pharmaceutics13070929

**Published:** 2021-06-23

**Authors:** Antonella Grigoletto, Gabriele Martinez, Daniela Gabbia, Tommaso Tedeschini, Michela Scaffidi, Sara De Martin, Gianfranco Pasut

**Affiliations:** Pharmaceutical and Pharmacological Sciences Department, University of Padua, Via F. Marzolo 5, 35131 Padova, Italy; antonella.grigoletto@unipd.it (A.G.); gabriele.martinez8740@gmail.com (G.M.); daniela.gabbia@unipd.it (D.G.); tommaso.tedeschini@unipd.it (T.T.); michela.scaffidi@unipd.it (M.S.)

**Keywords:** drug delivery, polymer conjugates, anticancer therapy, active targeting, paclitaxel, PEGylation

## Abstract

Although selective tumor delivery of anticancer drugs has been sought by exploiting either passive targeting or by ligand-mediated targeting, a selective anticancer therapy remains an unmet medical need. Despite the advances which have been achieved by nanomedicines, nanosystems such as polymer-drug conjugates still miss the goal of clinical efficacy. In this study, we demonstrated that polymer-drug conjugates require a thoroughly chemical design and the right targeting agent/polymer ratio to be selective and effective towards cancer cells. In particular, two PEG conjugates carrying paclitaxel and targeted with different folic acid (FA)/PEG ratios (one or three) were investigated. The cytotoxicity study in positive (HT-29) and negative (HCT-15) FA receptor (FR)-cell lines demonstrated that the conjugates with one or three FAs were 4- or 28-fold more active in HT-29 cells, respectively. The higher activity of the 3-FA conjugate was confirmed by its strong impact on cell cycle arrest. Furthermore, FA targeting had a clear effect on migration and invasiveness of HT-29 cells, which were significantly reduced by both conjugates. Interestingly, the 3-FA conjugate showed also an improved pharmacokinetic profile in mice. The results of this study indicate that thorough investigations are needed to optimize and tune drug delivery and achieve the desired selectivity and activity towards cancer cells.

## 1. Introduction

A variety of cytotoxic drugs have long been used in cancer chemotherapy, in single or combined protocols, with the aim of hitting and killing tumor cells while sparing healthy cells. Tumor selectivity with most of these drugs has been sought through preferential cytotoxicity against cells with higher proliferation or metabolic rates. However, these conditions are typical features of tumor cells but also many healthy cells, such as those of the bone marrow, digestive system epithelium and hair follicles. Furthermore, most existing anticancer drugs enter cells through non-specific lipophilic interactions with the cell membrane. Therefore, the therapeutic index of anticancer drugs is usually very low, since remarkable toxic side effects might compromise the therapeutic outcome.

The selective delivery of cytotoxic drugs to tumor cells have been extensively studied in an attempt to accumulate the payload preferentially in tumor tissues by exploiting the conjugation of targeting agents directly to these drugs or to the delivery systems carrying the drugs. This approach of ligand-mediated targeting is intended for improving the internalization of targeted nanomedicines by cells overexpressing a specific receptor. At the same time, it is important to increase the fraction of an administered nanomedicine that reaches the target site by exploiting the enhanced permeability and retention (EPR) effect, a feature typical of solid cancers [1]. Although both ligand-mediated targeting and the EPR effect have shown controversial results, which have not yet fulfilled the promise of a selective cancer therapy, their exploitation must be pursued and developed to reduce the toxic side effects of chemotherapies [2]. Antibody drug conjugates (ADCs) can be cited as an example of a targeted nanomedicine that can exploit both targeting approaches with great therapeutic outcomes [3]. Nevertheless, for ADCs the progress from the bench to the clinic is not easy [4] and the cost of such drugs might represent a limitation.

Polymer-drug conjugates have been studied by several researchers who obtained interesting results that unfortunately have not yet been translated into a clinical approved derivative. Most of the advanced conjugates investigated in clinical trials were relying only on the EPR effect for cancer therapy but did not achieve the desired selective cytotoxicity. During the years, researchers have learned about the limitations of the first conjugates, such as the difficulties of batch-to-batch reproducibility and the inadequacy or absence of proper ligand-mediated targeting. Still, such a drug delivery system offers unique properties, such as good water solubility of hydrophobic drugs, high drug payload and tunable physicochemical features.

To further investigate the potential of this delivery approach, the synthesis of macromolecular prodrugs is presented here. Poly(ethylene glycol) (PEG) was chosen as a polymeric carrier owing to its known biocompatibility, stability and solubility [5]. Heterobifuctional PEGs, eventually modified with a dendron structure, have the advantage to be suited for a defined structure design of the conjugates in which the targeting moiety is spatially separated from the drug moiety, thus generating a homogeneous structure that can overcome some limitations of random conjugates, as we have shown in other studies [6,7,8,9]. To further increase the selectivity of these PEG conjugates, the role of an active targeting agent was also investigated by linking folic acid (FA) to a heterobifunctional PEG. Paclitaxel (PTX) was used as the model drug.

The FA receptor is overexpressed in several types of human cancers, especially ovarian, but also kidney, uterus, brain, colon and lung cancer [10]. The folate receptor (FR) participates in the cellular accumulation of folate through the process of potocytosis, and is present as three different isoforms, i.e., two glycosylphosphatidylinositol-anchored membrane proteins, FR-α and FR-β, and a soluble form called FR-γ [11,12]. However, these receptors are expressed in the choroid plexus and placenta, and not in other healthy tissues, except for the lung, thyroid and kidney, where their expression is low. From a functional point of view, the α isoform of FR (FRα) has been linked to proliferation, migration, and invasion of tumor cells [13], and its overexpression has been demonstrated in approximately 40% of human colorectal carcinomas [14] and is associated with a worse prognosis [15]. Interestingly, FRα expression was also demonstrated in a subset of colorectal cancer liver metastases and has been independently associated with early death after hepatic resection [16], thereby confirming the peculiar role of this receptor not only in primary tumors but also in the metastasis process.

In light of these considerations and taking into account that FRα has been pointed to as a promising target for colon cancer-targeted therapy, we studied two PTX-PEG conjugates targeted with different amounts of FA and compared them with non-targeted conjugates. We could identify a lead conjugate characterized by a promising cytotoxic and antimetastatic activity in vitro and an improved pharmacokinetic profile in mice.

## 2. Materials and Methods

### 2.1. Materials

PTX was from LC Laboratories (Woburn, MA, USA). Boc-NH-PEG-NHS (5 kDa) and MeO-PEG-NH_2_ (5 kDa) were obtained from Iris Biotech GmbH (Marktredwitz, Germany). Folic acid (FA), *N*-Boc-1,5-diaminopentane, *N,N*-dicyclohexylcarbodiimmide (DCC), N-Hydroxysuccinimide (NHS), ethyl-3-(3-(dimethylamino)propyl)carbodiimide (EDC), 1-hydroxybenzotriazole (HOBT), succinic anhydride, silica gel (SiO_2_), trifluoroacetic acid (TFA), triethylamine (Et_3_N), 2,4,6-trinitrobenzenesulfonic acid (TNBS), dimethylsulfoxide-d_6_, methanol-d_4_, chloroform-d and all other chemical reagents including salts and solvents were purchased from Merk (Darmstadt, Germany).

### 2.2. Methods

Analytical RP-HPLC: analytical reverse phase (RP) HPLC was performed on an Agilent 1200 Series HPLC with online UV detection from Agilent Technologies (Santa Clara, CA, USA), using a Jupiter C18 column (4.6 × 250 mm; 5 μm; Phenomenex, Castel Maggiore, Italy) operating at a flow rate of 1 mL/min. Elution experiments were conducted with H2O + 0.05% TFA (eluent A) and ACN + 0.05% TFA (eluent B) (gradient B%: 0 min 5%, 25 min 40%, 30 min 70%, 35 min 95%, 40 min 5% B).

Mass spectrometry analysis: mass spectrometry-based analyses of FA derivatives and SPTX were performed with a mass spectrometer Xevo G2-S Q-Tof (Waters, Milford, MA, USA) equipped with an electrospray source. Samples were analyzed in MS positive ion mode. Measurements were conducted at a capillary voltage of 3 kV and at cone and extractor voltages of 35 and 1 V, respectively. Instrument control and data acquisition and processing were achieved with Masslynx software (version 4.1, Micromass, Santa Clara, CA, USA).

Mass spectra of PEG-FA were obtained using a REFLEX time-of-flight instrument (4800 Plus MALDI TOF/TOF, AB Sciex, Framingham, MA, USA) equipped with a SCOUT ion source, operating in the positive linear mode. A pulsed UV laser beam (nitrogen laser, λ 337 nm) generated ions that were accelerated to 25 kV. The matrix, a saturated solution of sinapinic acid in water/ACN (1:1, *v*/*v*) + 0.1% TFA (*v*/*v*), was mixed with an equal volume of sample solution and 1–2 μL were loaded on the plate.

NMR analysis: all ^1^H-NMR spectra were recorded on a Bruker 400 MHz spectrometer (Bruker, Billerica, MA, USA) with TMS as the internal standard.

### 2.3. Synthesis of PTX-PEG, PTX-PEG-FA and PTX-PEG-(FA)_3_ Conjugates

Preparation of FA-DAP-NH_2_: 1 g of FA (2.26 mmol) was solubilized at 40 °C in anhydrous DMSO. Next, 234 mg of DCC (0.5 eq, 1.13 mmol) was added and, after 30 min, 943 µL of *N*-Boc-1,5-diaminopentane (2 eq, 4.53 mmol, d = 0.972 g/mL) was added to the solution and the reaction was stirred at room temperature for 24 h. The reaction was monitored by RP-HPLC using the conditions reported above and the UV detector (Agilent Technologies, Santa Clara, CA, USA) settled at 280 nm. The mixture was filtered and added dropwise into a diethyl ether/THF 1:1 (*v*/*v*) solution. The precipitate was recovered by filtration, washed with a diethyl ether/THF 1:1 (*v*/*v*) mixture and dried under vacuum (yield: 1.2 g, 86.3% *w*/*w*). The product was characterized by ESI-TOF mass spectrometry (Waters, Milford, MA, USA). ESI-MS [*m*/*z*]: 626.29 (M + H)^+^ [Theorical mass: 625.29 Da].

Next, 1.2 g of Boc-DAP-FA (1.92 mmol) was dissolved in 25 mL of DMSO/CF_3_COOH 1:2 (*v*/*v*) mixture for 2 h to remove the protecting group t-Boc. After monitoring the reaction by RP-HPLC, as reported above, the mixture was evaporated to remove TFA and the obtained oil was dropped into a diethyl ether/THF 1:1 (*v*/*v*) mixture. The product was recovered by filtration, washed with a diethyl ether/THF 1:1 (*v*/*v*) mixture and dried under vacuum (yield: 1 g, 98.25% *w*/*w*). The intermediate FA-DAP-NH_2_ was characterized through ESI-TOF mass spectrometry. ESI-MS [*m*/*z*]: 526.28 (M + H)^+^ [Theorical mass: 525.24 Da].

Preparation of H_2_N-PEG-FA and H_2_N-PEG-(FA)_3_: the intermediates Boc-NH-PEG-FA and Boc-NH-PEG-(FA)_3_ were synthesized and purified using the same procedure. Here we report the procedure for Boc-NH-PEG-(FA)_3_ as an example. The dendron structure Boc-NH-PEG-(NHS)_5_ was prepared as described elsewhere [17,18]. Briefly, to 1 g of FA-DAP-NH_2_ (12 eq, 1.9 mmol) previously solubilized in 6 mL of DMSO at 40 °C, 48 µL of Et_3_N (0.34 mmol) and 600 mg of Boc-NH-PEG-(NHS)_5_ (0.11 mmol) were added. The mixture was left to react for 24 h at room temperature. The intermediate was purified by chromatography on a SiO_2_ column (30 × 2.5 cm) eluted with a chloroform/ethanol/acetone/ammonia mixture (2:2:0.5:0.25 to 2:2:1:0.5) and determined by RP-HPLC using the same conditions reported above with the UV detector settled at 280 nm. The purified product was concentrated under vacuum, solubilized in 1 mL of CH_2_Cl_2_ and dropped in cold diethyl ether. The precipitate was left for 1 h at −20 °C, recovered by centrifugation and dried under vacuum (yield: 293 mg, 38% *w*/*w*). The modification with FA was verified by ^1^H-NMR and MALDI-TOF.

Following this, 290 mg of Boc-NH-PEG-(FA)_3_ was dissolved in a CH_2_Cl_2_/CF_3_COOH/H_2_O (54:45:1) mixture for 3 h to remove the protecting group t-Boc. The solution was evaporated to remove the TFA and the obtained oil was solubilized in CH_2_Cl_2_ and dropped in cold diethyl ether. The product was recovered by centrifugation, dried under vacuum (yield: 276 mg, 95% *w*/*w*) and characterized through ^1^H-NMR.

Synthesis of 2′-succinyl-paclitaxel (SPTX): 293 mg (5 eq, 2.93 mmol) of succinic anhydride was added to 500 mg (0.59 mmol) of PTX, dissolved in 15 mL of anhydrous pyridine. The reaction was stirred at room temperature for 48 h. The SPTX was purified by chromatography on a SiO2 column (30 × 2.5 cm) eluted with a chloroform/methanol mixture (97:3 to 90:10) and determined by thin-layer chromatography (TLC; Rf 0.5 in chloroform/methanol, 90:10). The product yield was 97% (*w*/*w*). SPTX was characterized through ESI-TOF mass spectrometry and ^1^H-NMR. ESI-MS [*m*/*z*]: 954.46 (M + H)^+^ [Theorical mass: 953.35 Da]. ^1^H-NMR: (CD_3_OD, δ ppm), 1.15 (s, 3H, C16), 1.24 (s, 3H, C17), 1.68 (s, 3H, C18), 1.79 (s, 3H, C19), 2.24 (s, 3H, C31), 2.38 (s, 3H, C29), 2.5–2.7 (m, 4H, –CH_2_–CH_2_–succinic spacer), 4.9 (d, 1H, C5), 5.66 (d, 1H, C2′), 6.27 (s, 1H, C10), 7.25 (s, 3′-Ph), 7.4 (m, 3′-NBz), 7.5 (m, 2-OBz), 7.75 (d, 3′NBz), 8.1 (d, 2-OBz).

Synthesis of PTX-PEG, PTX-PEG-FA and PTX-PEG-(FA)_3_ conjugates: the conjugates were synthesized and purified using the same procedure. The preparation of PTX-PEG-(FA)_3_ is here described as an example. To 61.8 mg of SPTX (2 eq, 0.065 mmol) dissolved in anhydrous DMF, 13.7 mg (1.1 eq, 0.071 mmol) of EDC and 13.1 mg (1.5 eq, 0.097 mmol) of HOBT, previously dissolved in anhydrous DMF, were added. After 5 h under stirring at room temperature, H_2_N-PEG-(FA)_3_ (220 mg), previously dissolved in DMF, was added (PEG/SPTX ratio 1:2) and let to react for 48 h. Then DMF was removed under vacuum and the residue was dissolved in an ammonium acetate buffer of 10 mM, pH 4.7. The excess of SPTX was eliminated by extractions with diethyl ether and the solution was dialyzed against ammonium acetate buffer 10 mM, pH 4.7 before lyophilization. The product was characterized by ^1^H-NMR and RP-HPLC. The content of free PTX was evaluated by RP-HPLC, preparing conjugates solutions at the concentration of 5 mg/mL in methanol. The RP-HPLC conditions were the same as reported above with the UV detector settled at 227 nm.

### 2.4. Determination of Conjugated PTX

The content of the conjugated drug was evaluated by RP-HPLC after freeing PTX. The solutions of conjugates and PTX (for the calibration curve) were prepared in methanol and underwent the same treatment. Following the addition of 2% (*v*/*v*) of 0.2 N NaOH, the solutions were incubated at 50 °C for 2 h. The drug was extracted by ethyl acetate and after evaporation of the organic phase, the residue was solubilized in methanol and analyzed in RP-HPLC, as reported above. The elution was performed using a Jupiter C18 column (21.2 × 250 mm; 5 μm; Phenomenex), eluted with H_2_O + 0.05% TFA (eluent A) and MeCN + 0.05% TFA (eluent B) at 1.0 mL/min flow rate (gradient B%: 0′ 5%, 25′ 40%, 30′ 70%, 35′ 95%, 40′ 5% B). The UV detector was settled at 227 nm. The amount of conjugated PTX was calculated using a calibration curve of drug solutions at known concentrations.

### 2.5. Dynamic Light Scattering (DLS) Analysis

The mean hydrodynamic diameter and the stability of polymeric structures of PTX-PEG, PTX-PEG-FA and PTX-PEG-(FA)_3_ conjugates were analyzed using a light scattering instrument (Malvern Zetasizer Nano ZS, Malvern, UK). To evaluate the stability of the conjugates, solutions of each compound (7 mg/mL) were prepared in PBS at pH 7.4 and the size of the micelles was monitored every 20 min for 6 h and at 24 h. The instrument was settled at 37 °C.

### 2.6. Cell Cultures

The colorectal cancer cell lines HT-29 (FR-positive) and HCT-15 (FR-negative) were a kind gift from Professor Antonio Rosato (University of Padova, Padova, Italy). Cell were maintained at 37 °C in standard CO_2_ (5%) and humidity conditions, in the RPMI medium with the addition of 10% foetal bovine serum (FBS), 1% HEPES, 1% glutamine, 1% Penicillin Streptomycin (all purchased from Corning, Corning, NY, USA).

### 2.7. Immunocytochemistry

To assess the FR expression in HT-29 and HCT-15 cells, we performed an immunofluorescence analysis coupled to confocal microscopy [19]. Briefly, cells were seeded into glass coverslip-coated plates (20,000 cells/mL), fixed for 20 min with 4% PFA at room temperature and incubated for 10 min with 5% FBS to block unspecific cross-reaction. After the PBS wash, cells were incubated overnight at 4 °C with a primary antibody directed against the folate receptor (dilution 1:50, sc-515521 Santa Cruz), and then at 37 °C for 1 h with an anti-mouse Alexa Fluor 568-conjugated secondary antibody (dilution 1:500). To counterstain cell nuclei, the reagent Hoechst 33342 was used (Thermo Scientific, Waltham, MA, USA). Cell images were acquired by means of a confocal microscope Zeiss LSM 800 (Zeiss, Milan, Italy) and analyzed with the ImageJ software (ver. 1.52, developed by NIH, Bethesda, MD, USA).

### 2.8. Cell Viability Studies

Cell viability was assessed by the ATPlite (Perkin-Elmer Inc., Waltham, MA, USA) kit, following the manufacturer’s instructions. Briefly, HCT-15 and HT-29 cells were seeded (3000 cells/well) in a 96-well plate [19]. After 24 h, cells were incubated for 48 h with increasing concentrations of PTX and PTX conjugates (range: 0.0125 pg/mL–50 mg/mL, PTX equiv.). At the end of the incubation period, luminescence was measured by the multiplate reader Victor Nivo (Perkin-Elmer Inc., Waltham, MA, USA). The 50% inhibitory concentrations (IC_50_) were calculated by the appropriate nonlinear regression fit, using the GraphPad Prism software (San Diego, CA, USA), version 8.0.

### 2.9. Cell Cycle Analysis

A cell cycle analysis was performed as already described [20]. Briefly, HT-29 and HCT15 cells (60,000 cells/mL) were seeded and treated with PTX and PTX conjugates (half IC_50_). After 24 h, cells were fixed with 70% ethanol at 4 °C for 15 min. After fixation, cells were recovered by centrifugation and stained with 350 µL PBS containing propidium iodide (0.07 mg/mL) and RNAse (0.7 mg/mL) for 15 min in the dark. Stained cells were analyzed by means of the Epics XL flow cytometer (Beckman Coulter, San Diego, CA, USA) using the CXP software (version 2.2, Beckmann Coulter, San Diego, CA, USA).

### 2.10. Wound Healing Assay

A wound healing assay was performed to analyze cell migration ability, as described before in [19]. Briefly, 10,000 cells were left adhered into 12-well plates for 48 h. After seeding, a scratch in the cell monolayer was made using a pipette tip and washed with PBS. Cells were then treated with PTX and PTX conjugates (half IC_50_) for 24 h. Cell migration in the wound area was assessed by means of a microscope (10× magnification) and Image J software. The percentage of scratch closure was calculated using the following Equation (1):

(1) $$Wound\;closure\;\left(\%\right)=\frac{Wound\;area_{0}-Wound\;area_{24}}{Wound\;area_{0}}\times 100$$

### 2.11. mRNA Expression Analysis by qRT-PCR

To perform mRNA expression analysis, HT-29 and HCT-15 cells (15 × 10^4^ cell/well) were seeded into 6-well plates. After 24 h, cells were treated with PTX and PTX conjugates (half IC_50_) for 24 h. Total mRNA was extracted from cells by means of the TRIzol and Direct-zol RNA MiniPrep (Zymo Research, Irvine, CA, USA), following the manufacturers’ instructions. qRT-PCR was performed by a QuantiNova SYBR Green RT-PCR Kit (Germantown, MD, USA) on an Eco Illumina Real-Time PCR system (Illumina Inc., San Diego, CA, USA) [21]. The sequences of the primers used in this study and their main characteristics are reported in Table 1. Relative mRNA quantification was obtained according to the ∆∆Ct method, using GAPDH as the housekeeping gene [22].

### 2.12. ELISA Assay for VEGF Quantification

VEGF release was measured in cell supernatants after 24 of treatment with PTX and PTX conjugates (half IC_50_) by means of a commercially available ELISA kit (ELH-VEGF, Raybiotech, Peachtree Corners, GA, USA), following the manufacturer’s instructions. At the end of the procedure, the plates were read at 450 nm with multi-plate reader Victor Nivo (Perkin-Elmer Inc., Waltham, MA, USA), and the VEGF concentration was calculated from calibration curves plotted on log-log graphs.

### 2.13. Pharmacokinetic Study in Mice

All the procedures involving animals were conducted in agreement with national and international regulations (Directive 2010/63/EU), and suitable procedures were taken to minimize animal pain or discomfort. The procedures were reviewed and approved by the Ethical Committee of the University of Padova (OPBA), and by the Italian Ministry of Health, in the section for the care and use of laboratory animals (authorization no. 938/2016-PR, obtained on August 4, 2016). The pharmacokinetic profiles of PTX-PEG, PTX-PEG-FA and PTX-PEG-(FA)_3_ were determined in female Balb/C mice (25–30 g). A total of 30 mice were randomly divided into 2 groups of 15 animals. The samples were solubilized in phosphate buffered saline (PBS) pH 6.2. A dose of 10 mg/kg (PTX equiv.) was administered via the tail vein to mice anaesthetized with 5% isoflurane gas mixed with O_2_ in enclosed cages. At predetermined times, blood samples were withdrawn from the submandibular plexus. The blood samples were centrifuged at 1500× *g* for 15 min. To 50 μL of plasma, 350 μL of ACN was added to obtain plasma proteins precipitation, and the resulting mixture was centrifuged at 20,000× *g* for 5 min. Furthermore, 300 μL of supernatant was collected and dried in speed-vac. The residue was dissolved in methanol and hydrolyzed with 0.2 NaOH as reported above. PTX plasma *concentration-vs-time* data were analyzed by means of the 2.0 PkSolver, an add-in software for pharmacokinetic analysis in Microsoft Excel (Redmond, WA, USA). A bi-exponential equation was the best-fitting equation for the pharmacokinetic data obtained in this study. The main pharmacokinetic parameters were calculated from the coefficients and exponents of the best-fits by using standard formulae [23].

### 2.14. Statistical Analysis

The results obtained in this study were analyzed by the GraphPad Prism software (San Diego, CA, USA), version 8.0 and compared by a Student’s T test or one-way ANOVA followed by the Tukey’s *post hoc* test, when appropriate. Unless otherwise stated, data are reported as mean ± S.E.M. *p* values < 0.05 were considered statistically significant.

## 3. Results

### 3.1. Synthesis and Characterizarion of PTX-PEG, PTX-PEG-FA and PTX-PEG-(FA)_3_ Conjugates

**Figure 1:**
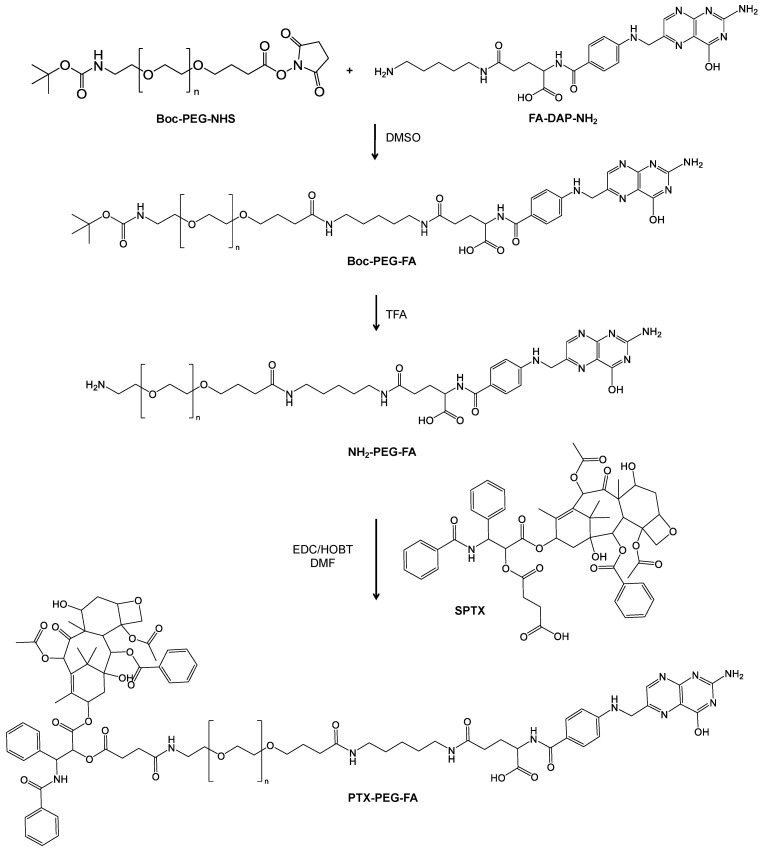
Synthesis of PTX-PEG-FA.

Two PEG conjugates carrying PTX and a different loading of FA were prepared starting from Boc-NH-PEG5k-NHS. The synthesis of the conjugates PTX-PEG-FA and PTX-PEG-(FA)_3_ was performed as reported in Figure 1 and Figure S1 and it required three main steps: the synthesis of PEG-FA, the succinylation of the PTX hydroxyl group in the 2′ position and the binding of SPTX to H_2_N-PEG-FA or H_2_N-PEG-(FA)_3_.

The modification of FA with N-Boc-1,5-diaminopentane was monitored by RP-HPLC and the formation of the mono-derivative of FA was verified through ESI-TOF mass spectrometry (Figure S2). The applied reaction conditions have avoided the derivatization of both carboxylic groups of FA, generating the undesired side product FA-(DAP-NH_2_)_2_. FA-DAP-NH_2_ formation was monitored by RP-HPLC and confirmed by ESI-TOF mass spectrometry (Figure S3). The collected intermediate was a mixture of free FA and FA-DAP-NH_2_, and the number of amino groups was calculated to be 64.5% on the basis of the Snaider–Sobocisky assay. Free FA could not react with Boc-PEG-NHS and consequently its removal was postponed after the synthesis of Boc-PEG-FA. The binding of FA-DAP-NH_2_ to PEG and the absence of free FA or FA-DAP-NH_2_ were checked by RP-HPLC. The MALDI-TOF analysis also confirmed the formation of PEG-FA derivatives (Figures S4 and S5). After purification, the products were characterized by ^1^H-NMR (Figure S6 panel A and Figure S7 panel A). The linear derivative Boc-PEG-FA showed the conjugation with one molecule of FA, on the basis of ^1^H-NMR evaluation, while the coupling of the PEG dendron to FA-DAP-NH_2_ resulted in about three molecules of FA for the PEG unit (Table 1). The modification of PTX with the succinic spacer was verified by ESI-TOF mass spectrometry (Figure S8) and ^1^H-NMR (Figure S9). After the removal of the Boc protecting group from PEG-FA intermediates (Figure S6 panel B and Figure S7 panel B), SPTX was conjugated to the free amino group of H_2_N-PEG-FA and H_2_N-PEG-(FA)_3_ via activation of the carboxylic group of the succinic spacer. Purified PTX-PEG-FA and PTX-PEG-(FA)_3_ were characterized by ^1^H-NMR (Figure S6 panel C and Figure S7 panel C) showing the characteristic signals of PTX together with those of the succinic spacer, PEG and folic acid. The synthesis and the characterization of non-targeted PTX-PEG were reported in Supplementary Materials (Figures S10 and S11). The reaction yields of SPTX coupling were similar for all the conjugates and are listed in Table 1. The content of free drugs in PTX-PEG, PTX-PEG-FA and PTX-PEG-(FA)_3_ was found to be less than 1.5% with respect to the total drug amounts, as determined by RP-HPLC. The loading *w*/*w*% of PTX of each conjugate is reported in Table 2.

**Figure 2:**
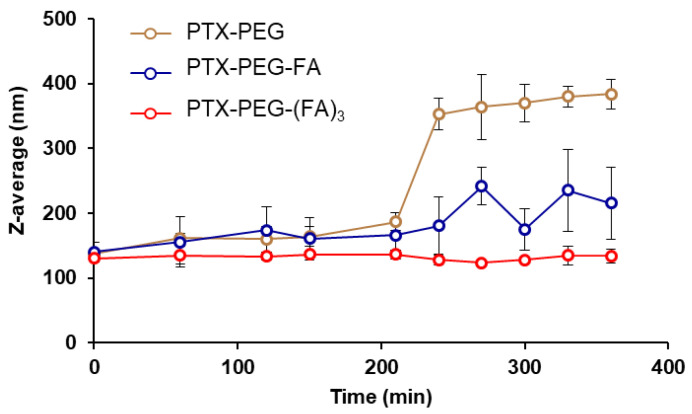
Evaluation of PTX-PEG, PTX-PEG-FA and PTX-PEG-(FA)_3_ nanoassemblies stability in PBS buffer at pH 7.4, 37 °C. The size of the micelles was monitored using by DLS.

Beyond the chemical structure and the control of the composition, another important aspect is the conformation of the conjugates in a solution that may affect body biodistribution and clearance. Being amphiphilic, an FA-targeted PEG-PTX conjugate can arrange supramolecular nanoassemblies as shown by DLS measurements (Table 1), with sizes in the 130–140 nm range (Figure S12). The stability of PEG-PTX conjugate nanoassemblies in the solution was monitored by DLS at 37 °C for up to 24 h. PTX was linked to the polymer through an ester linkage which undergoes hydrolysis, thus releasing the drug. The size of PTX-PEG and PTX-PEG-FA nanoassemblies started to increase after about four hours of incubation as a consequence of PTX released from the conjugates, which destabilized the system, while the micelles of PTX-PEG-(FA)_3_ were stable for a prolonged time (Figure 2). For PTX-PEG-(FA)_3_, the structure of the micelles was maintained up to 24 h, suggesting a slower hydrolysis rate of the drug. We could speculate that despite the fact that a certain degree of PTX was hydrolyzed, such PTX molecules were retained in the inner core of the nanoassemblies, due to the high hydrophobicity of the drug.

### 3.2. PTX-PEG-FA Conjugates Induced Cytotoxicity on FR-Postive HT-29 Cells

**Figure 3:**
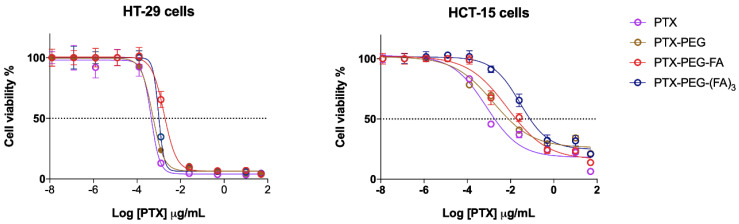
Cytotoxicity of PTX and PTX conjugates in FR-positive (HT-29) and FR-negative (HCT-15) cells. The results are the mean of three independent experiments, each performed in triplicate.

**Figure 4:**
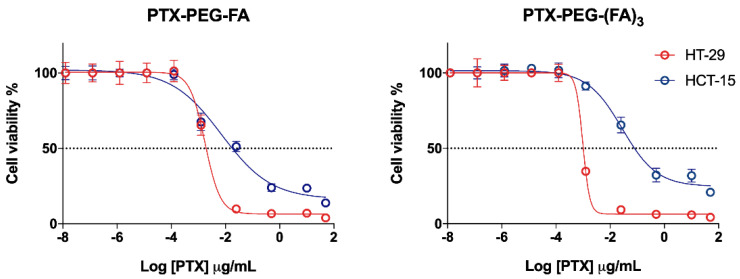
Graphical comparison between the cytotoxicity of the two targeted conjugates in FR-positive (red) and FR-negative (blue) cells.

The conjugates were tested in two CRC cell lines with different expressions of the FA receptor (Figure S13). In FR-positive HT-29 cells, both the targeted PTX conjugates were more active compared to FR-negative HCT-15 cells, whereas PTX and PTX-PEG-induced cytotoxicity was similar in the two cell lines (Figure 3). However, a slight increase (*p* < 0.05) of PTX-PEG IC_50_ values could be observed in HCT-15 cells with respect to HT-29. Furthermore, in HT-29 cells the cytotoxic activity of the PTX-PEG-(FA)_3_ conjugate was similar to that of unconjugated PTX and PTX-PEG, whereas a slight but significant (*p* < 0.05) drop of activity was observed for the PTX-PEG-FA conjugate with respect to PTX and the PTX-PEG conjugate (Figure 3, Table 3). However, no statistical difference could be observed between the activities of the two targeted conjugates in HT-29 cells (Figure 4). In HCT-15 cells, the conjugation of PTX caused a significant drop of its activity, especially for the PTX-PEG-(FA)_3_ conjugate, which was less cytotoxic not only than unconjugated PTX and PTX-PEG (*p* < 0.0001), but also than PTX-PEG-FA (*p* < 0.001).

### 3.3. PTX-PEG-(FA)_3_ Induces G1/M Phase Cell Cycle Arrest in HT-29 Cells

**Figure 5:**
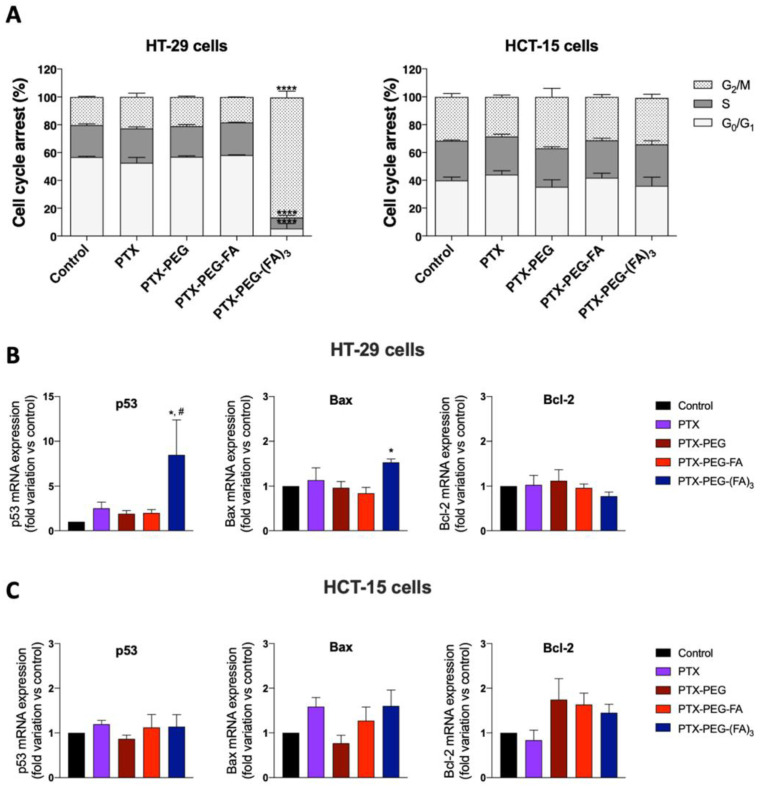
(**A**) Cell cycle distribution of HT-29 and HCT-15 cells exposed to PTX and PTX conjugates. **** p < 0.0001 vs. all the other treatments. Effect of PTX and PTX conjugates on the mRNA expression of the pro-apoptotic genes p53 and Bax, and the anti-apoptotic gene Bcl-2 on HT-29 (**B**) and HCT-15 (**C**) cells. * *p* < 0.05 vs. control, ^#^ *p* < 0.05 vs. cells treated with PTX. The results are the mean of three independent experiments, each performed in triplicate.

In order to obtain additional information about PTX-PEG-FA conjugates on HT-29 and HCT-15 cells, we investigated by flow cytometry the cell cycle distribution after 24 h of treatment at the half IC_50_ values reported in Table 3. As shown in Figure 5A, the percentage of HT-29 cells in G2/M phase were dramatically increased only by the PTX-PEG-(FA)_3_ conjugate in these experimental conditions, from 20% of the control to 85% for HT-29 cells treated with this conjugate. Conversely, no effect was observed in HCT-15 cells. These findings indicate that PTX-PEG-(FA)_3_ displays a peculiar cytotoxicity towards FR-positive HT-29 cells, by arresting the cell cycle in the G2/M phase. Accordingly, only PTX-PEG-(FA)_3_ caused a significant increase of the mRNA expression of the two pro-apoptotic genes p53 and Bax, and a decreasing tendency of the anti-apoptotic gene Bcl-2. This effect was not observed in HCT-15 cells for which, in accordance with the data about cell cycle arrest, the treatments did not change the expression of the tested anti- and pro-apoptotic genes.

### 3.4. PTX-PEG-FA Conjugates Inhibit Migration of HT-29 Cells

**Figure 6:**
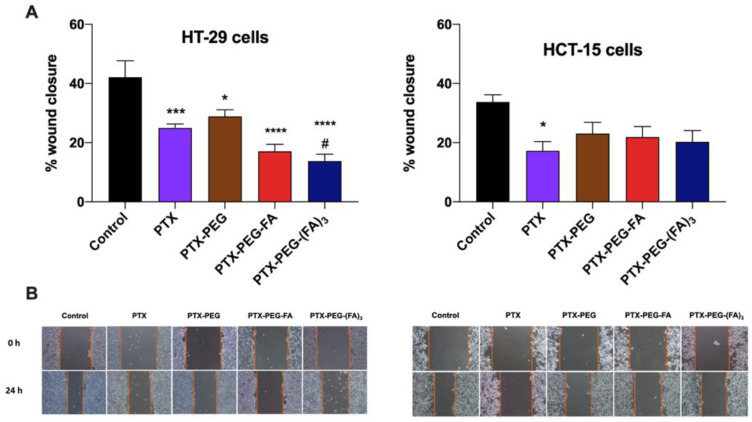
Wound healing assay in the CRC cell lines HT-29 and HCT-15. (**A**) Quantitative analysis of scratch wound healing assay after a 24 h treatment with NEM or CP (half IC_50_/48 h). (**B**) Representative images of the scratch wound healing (magnification 10×). The results are the mean of three independent experiments. * *p* < 0.05, *** *p* < 0.001, **** *p* < 0.0001 vs. untreated control cells; # *p* < 0.05 vs. cells treated with PTX.

Since the generation of metastasis is a peculiar feature of colorectal cancer [24], we performed a wound healing assay in HT-29 and HCT-15 cells treated with PTX or its conjugates (half IC_50_) for 24 h. In Figure 6, it is clearly shown that PTX and all the tested conjugates were able to reduce the migration of HT-29 cells. However, only PTX-PEG-(FA)_3_ was significantly more effective than PTX in reducing HT-29 migrative capacity in these experimental conditions. In contrast, only free PTX significantly reduced the migration of FR-negative HCT-15 cells.

**Figure 7:**
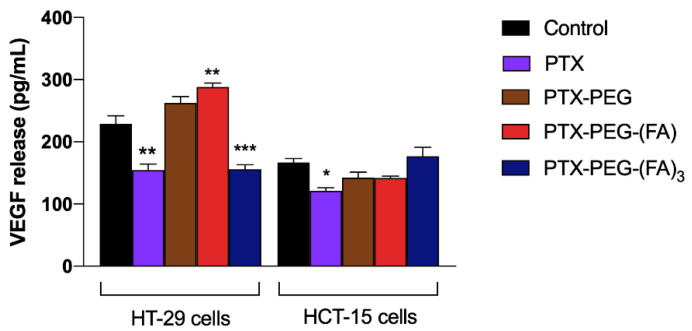
VEGF release from HT-29 and HCT-15 cells exposed to PTX and PTX-PEG-FA conjugates. * *p* < 0.05, ** *p* < 0.01, *** *p* < 0.001 vs. control untreated cells. The results are the mean of three independent experiments, each performed in duplicate.

To confirm the results of the wound healing assay, we measured the vascular endothelial growth factor (VEGF) release from HT-29 and HCT-15 cells in cell supernatants, because it is well-known that VEGF is a key player in the migration and invasion of cancer cells [25]. In Figure 7, it is shown that PTX and PTX-PEG-(FA)_3_ significantly reduced VEGF release from HT-29 cells, confirming their inhibiting effect on the migration of FR-positive cells, whereas VEGF release from FR-negative cell migration was reduced only by free PTX.

**Figure 8:**
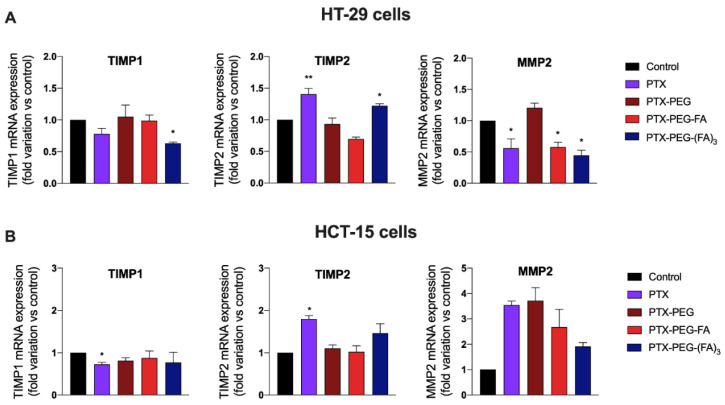
Effect of PTX and PTX conjugates on the mRNA expression of the TIMP1, TIMP2 and MMP2 on HT-29 (**A**) and HCT-15 (**B**) cells. * *p* < 0.05, ** *p* < 0.01, vs. control cells. The results are the mean of three independent experiments, each performed in triplicate.

Finally, since invasion and metastasis are processes modulated by matrix metalloproteases (MMP) and their inhibitors TIMPs, we analyzed the effect of PTX and its conjugates on the mRNA expression of three genes known to participate in the migration and invasiveness of colon cancer cells., i.e., TIMP1, TIMP2 and MMP2 [26]. Figure 8 shows that PTX-conjugates can upregulate the mRNA expression of TIMP2 and downregulate that of TIMP1 and MMP2 only in FA-positive HT-29 cells. This effect on genes involved in migration and invasiveness is more evident in cells treated with the conjugate PTX-PEG-(FA)_3_.

### 3.5. Pharmacokinetic Studies in Mice

**Figure 9:**
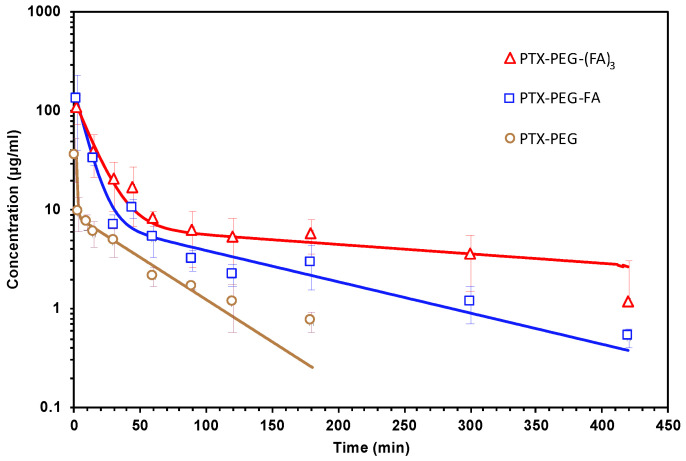
Pharmacokinetic profiles of PTX-PEG-FA and PTX-PEG-(FA)_3_ in Balb/C mice after IV administration of 10 mg/kg (PTX equiv.).

The pharmacokinetics of PTX-PEG, PTX-PEG-FA and PTX-PEG-(FA)_3_ were studied in Balb/C mice after IV administration (Figure 9, Table 4). The two FA-targeted PTX conjugates showed a significant half-life prolongation with respect to that of PTX. For the pharmacokinetic profile of PTX we refer to our previous study in which the PTX plasma concentration fell below the limit of detection after 30 min post injection with a t_½β_ of 15 min [8]. In particular, PTX-PEG-(FA)_3_ exhibited a great increase of the t_½β_, reaching a value of about 5 h, and caused an increase of PTX bioavailability, since its AUC showed an approximate 2-fold increase with respect to that of the linear targeted derivative.

## 4. Discussion

Targeted drug delivery systems have been proposed as a key strategy for achieving the selective transport of cytotoxic cargos from the site of injection to the cancer cells. Ligand-mediated targeting alone might not always be sufficient for a targeted delivery of proper amounts of drugs at the site of action, and neither is the EPR effect alone for those nanomedicines that focus only on the increment of sizes for exploiting passive targeting. Consequently, to maximize as much as possible the effectiveness of these drug delivery systems, joining the positive features of both targeting strategies, commonly defined as active and passive targeting, is necessary. Here we seek to investigate this possibility by studying polymeric drug conjugates targeted with FA for active targeting, which showed an increased size in solution for a better pharmacokinetic and the desired tumor accumulation through the EPR effect.

At the basis of these FA-targeted conjugates there is a heterobifunctional PEG that presents two different reactive groups at the chain ends, thus offering the possibility of coupling at separate sites the drug and the targeting agent for a defined chemical structure of the conjugates. The FA-targeted PEG-PTX conjugates were developed with a precise composition to allow the investigation of the role of each component in terms of activity with respect to cell lines with different expression levels of the target, i.e., the FA receptor (Figure S11). PEG with a molecular weight of 5kDa was selected because such size ensures the solubilization in water of the hydrophobic PTX and allows the formation of nanoassemblies in the solution, as we already reported with alendronate as targeting agent [8]. The FA-targeted PEG-PTX conjugate with an increased payload of FA was prepared by synthetizing a dendron structure based on β-glutamic acid as the branching unit, with the aim of improving the targeting efficiency and selectivity of the conjugate towards the cancer cells overexpressing the FA receptor. The synthesis of a dendron structure offers the possibility to design a polymer drug conjugate with the desired targeting characteristics. FA was linked to carboxylic units of PEG through the 1,5-diaminopentane spacer, to increase the mobility of FA and favor the binding with its receptor.

The conjugates were obtained in good yields and resulted in completely soluble aqueous buffers, contrary to PTX that, in clinical use, needs to be solubilized in the hydrophobic vehicle Cremophor-EL. PTX-PEG, PTX-PEG-FA and PTX-PEG-(FA)_3_ were tested in two human colorectal adenocarcinoma cell lines with different expressions of the FA receptor for evaluating whether the increased payload of the targeting agent in PTX-PEG-(FA)_3_ could have an impact on the cytotoxic activity. IC_50_ values (Table 2) showed that overall FA-targeted PEG-PTX conjugates were more active in FR-positive HT-29 cells than in FR-negative HCT-15 cells. This result positively confirmed that these conjugates can be selectively toxic towards target cells while at the same time sparing the cells not expressing the target. Furthermore, by comparing the two FA-targeted PEG-PTX conjugates we can affirm that the selective cytotoxicity towards target cells (HT-29) with respect to the cytotoxicity in non-target cells (HTC-15) was augmented by increasing the number of targeting molecules per polymer chain. In fact, PTX-PEG-FA was 4-fold more active in HT-29 compared to HCT-15 while PTX-PEG-(FA)_3_, under the same conditions, was more than 28-fold more cytotoxic. These ratios correspond to just 3-fold for PTX-PEG and less than 2-fold for PTX. Consequently, apart from the relevance of the presence of active targeting, this study also points to the targeting ligand payload with the polymer-drug conjugate. Furthermore, PTX-PEG-(FA)_3,_ besides increasing significantly PTX cytotoxicity due to the enhanced target capacity, showed a peculiar effect on the cell cycle, since it was able to arrest 85% of the cells in the G2/M phase. To confirm this observation, we performed a qPCR analysis to measure the expression of three genes involved in apoptosis, namely the pro-apoptotic p53 and Bax and the anti-apoptotic Bcl-2, thereby observing that only PTX-PEG-(FA)_3_ significantly affects their expression by increasing both p53 and Bax. Interestingly, the activation of p53 has been linked to the arrest of the cell cycle in the G2/M phase and to an enhancement of efficacy of antimitotic drugs including PTX [27]. It is possible to hypothesize that the targeting effect of the three FA molecules is particularly efficient and yielded a PTX concentration inside the cells, at the end of the incubation, which was sufficient to exert the observed effect on cell cycle distribution and mRNA expression [28].

In addition, we investigated whether the generation of these PTX-PEG-FA conjugates could have a specific effect on migration and invasiveness of FR-positive colorectal cancer cells. Interestingly, only PTX-PEG-(FA)_3_ could both reduce HT-29 cell migration, as demonstrated in the wound healing assays, and decrease the release of the pro-metastatic agent VEGF. A peculiar effect of PTX-PEG-(FA)_3_ was observed also on the mRNA expression of the tissue inhibitor of metalloproteinases (TIMP)1, a prognostic marker of progression and metastasis of colon cancer [29], whose mRNA expression was decreased only by this PTX conjugate. Based on their relevance in colorectal cancer invasion [26], we also evaluated the mRNA expression of the matrix metalloproteinase 2 (MMP2) and its inhibitor TIMP2. It has been demonstrated that MMP2 is upregulated in invasive colorectal cancers and participates in the metastatic process by degrading β1 integrins, thereby decreasing cell adhesion and enhancing their motility [30]. Accordingly, the reduced expression of the MMP2 inhibitor TIMP2 correlates with colorectal cancer invasion and a worse prognosis [31]. We found that PTX and PTX-PEG-(FA)_3_ can upregulate the mRNA expression of TIMP2 and downregulate that of MMP2, thereby confirming their effect on colon cancer cell migration and invasiveness. Furthermore, PTX-PEG-(FA)_3_ demonstrated the most promising features also in vivo since, as expected, it showed an improvement of the PTX pharmacokinetic profile which was superior to that obtained by the conjugate with only one FA molecule. This led to an increased and prolonged in vivo drug exposure and suggested a potential accumulation in tumors by the EPR effect.

## 5. Conclusions

The obtainment of a well-defined targeted polymer-drug conjugate in which the targeting moiety is spatially separated from the cytotoxic drug is not trivial and passes through an accurate drug design. Only an accurate study of each conjugate component will lead to the development of drug-polymer conjugates with improved and selective cytotoxicity.

## Figures and Tables

**Table 1:** qPCR primers used in this study.

Gene	Forward Sequence	Reverse Sequence	RefSeq	Product Length (Bp)
p53	GAGACCTGTGGGAAGCG	CGGGGACAGCATCAAAT	NM_001126118.2	123
Bax	CACTGAAGCGACTGATGTCCC	CCGCCACAAAGATGGTCAC	NM_001291428.2	91
Bcl2	TGTGTGTGGAGAGCGTCAA	CAGCCCAGACTCACATCACCA	NM_000657.3	148
TIMP-1	GCTGTGAGGAATGCACAGTGTTT	GGACTGGAAGCCCTTTTCAGA	NM_003254.3	116
TIMP-2	TCTGTGACTTCATCGTGCCC	ATGTAGCACGGGATCATGGG	NM_003255.5	121
MMP-2	CATCCAGACTTCCTCAGGCGG	GGTCCTGGCAATCCCTTTGTATG	NM_004530.6	75
GAPDH	ACATCAAGAAGGTGGTGAAGCA	GTCAAAGGTGGAGGAGTGGTT	NM_002046.7	119

**Table 2:** PTX-PEG, PTX-PEG-FA and PTX-PEG-(FA)3 reaction yields (*w*/*w*)%, loading% *w*/*w* of FA and PTX and mean hydrodynamic diameter of polymeric structure in PBS at pH 7.4.

Conjugate	% of Free PTX	FA Loading (mol%)	PTX Loading (*w*/*w*%)	Hydrodynamic Diameter (nm)
PTX-PEG	1.33	-	9.11 ± 0.1	137.4 ± 1.8
PTX-PEG-FA	1.47	1	10.27 ± 1.02	140.4 ± 0.18
PTX-PEG-(FA)_3_	0.79	3	8.53 ± 0.2	130.1 ± 2.28

**Table 3:** IC_50_ values (ng/mL PTX).

Cell Line	PTX	PTX-PEG	PTX-PEG-FA	PTX-PEG-(FA)_3_
HT-29	0.444 ± 0.123	0.518 ± 0.241	1.766 ± 0.791 ^#^	0.979 ± 0.151
HCT-15	0.750 ± 0.265	1.580 ± 0.454 *	7.153 ± 1.423 **^,#^	27.69 ± 4.12 ***^,####,§§§^

^#^*p* < 0.05 and ^####^ *p* < 0.0001 vs. PTX and PTX-PEG treatment in the same cell line; ^§§§^ *p* < 0.001 vs. PTX-PEG-FA treatment in the same cell line; * *p* = 0.0231 vs. HT-29 cells treated with PTX-PEG; ** *p* = 0.0017 vs. HT-29 cells treated with PTX-PEG-FA; *** *p* = 0.0004 vs. HT-29 cells treated with PTX-PEG-(FA)_3_.

**Table 4:** Main pharmacokinetic parameters of PTX-PEG-FA and PTX-PEG-(FA)_3_ after IV administration of 10 mg/kg (PTX equiv.) in Balb/C mice.

Sample	t_½α_ (min)	t_½β_ (min)	AUC 0-inf (µg min/mL)	CL (mL/min)	Vd (mL)
PTX-PEG	0.46	35.2	1566.3	0.15	6.6
PTX-PEG-FA	5.62	95.4	2438.5	0.12	8.2
PTX-PEG-(FA)_3_	8.94	313.8	4740.78	0.08	7.6

## Data Availability

Data are available upon reasonable request to the corresponding authors.

## Supplementary Materials

The following are available online at https://www.mdpi.com/article/10.3390/pharmaceutics13070929/s1: Figure S1: Scheme of synthesis of PTX-PEG-(FA)_3_; Figure S2: Mass spectrum of Boc-DAP-FA; Figure S3: Mass spectrum of FA-DAP-NH2; Figure S4: Mass spectrum of Boc-PEG-FA; Figure S5: Mass spectrum of Boc-PEG-FA_3_; Figure S6: 1H-NMR spectra of Boc-PEG-FA, H_2_N-PEG-FA and PTX-PEG-FA; Figure S7: Comparison of 1H-NMR of Boc-NH-PEG-(FA)_3_, H2N-PEG-(FA)_3_, and PTX-PEG-(FA)_3_; Figure S8: Mass spectrum of SPTX; Figure S9: 1H-NMR spectra of PTX and SPTX; Figure S10: Synthesis of PTX-PEG; Figure S11: 1H-NMR spectra of mPEG-NH_2_ (A) and PTX-PEG (B); Figure S12: Size distribution by DLS of PTX-PEG (A), PTX-PEG-FA (B) and PTX-PEG-(FA)_3_; Figure S13: Expression of Folic Acid receptor on HT-29 and HCT-15 cells.
