# Supplementary material for: Folic Acid-Targeted Paclitaxel-Polymer Conjugates Exert Selective Cytotoxicity and Modulate Invasiveness of Colon Cancer Cells
Source: Pharmaceutics. 2021 Jun 23;13(7):929. doi: 10.3390/pharmaceutics13070929 (PMC8309175; doi:10.3390/pharmaceutics13070929)
Supplement: Supplementary file 1 [file pharmaceutics-13-00929-s001.zip › pharmaceutics-1258228-supplementary.pdf]

pharmaceutics

MDPI

Supplementary Materials: Folic Acid-Targeted Paclitaxel-Polymer Conjugates Exert Selective Cytotoxicity and Modulate Invasiveness of Colon Cancer Cells

Antonella Grigoletto, Gabriele Martinez, Daniela Gabbia, Tommaso Tedeschini, Michela Scaffidi, Sara De Martin and Gianfranco Pasut

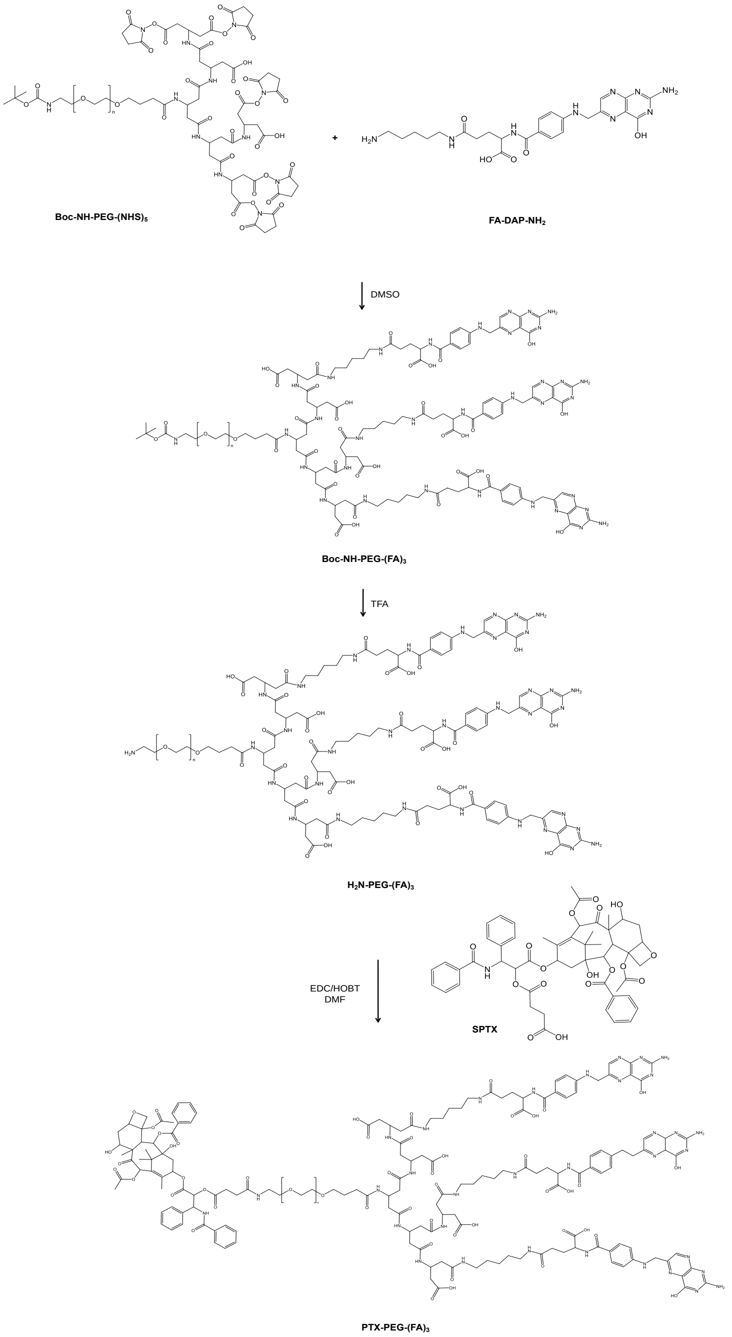

Figure S1. Synthesis of PTX-PEG-(FA)3. FA = folic acid, DAP = 1,5 di-amino pentane, DMSO = dimethylsulfoxide, EDC = 1-ethyl-3-(3-dimethylaminopropyl)carbodiimide, HOBT = hydroxybenzotriazole, SPTX = succinimidyl paclitaxel.

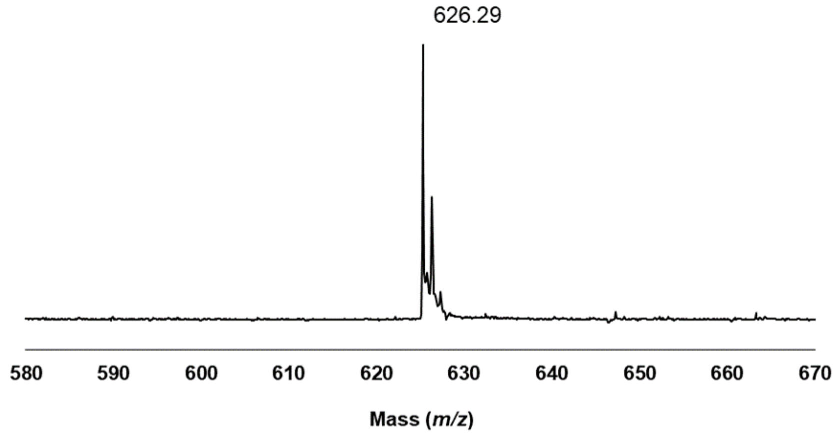

Figure S2. ESI-TOF mass spectrometry of Boc-DAP-FA. ESI-MS [*m*/*z*]: 626.29 (M + H)+ [Theorical mass: 625.29].

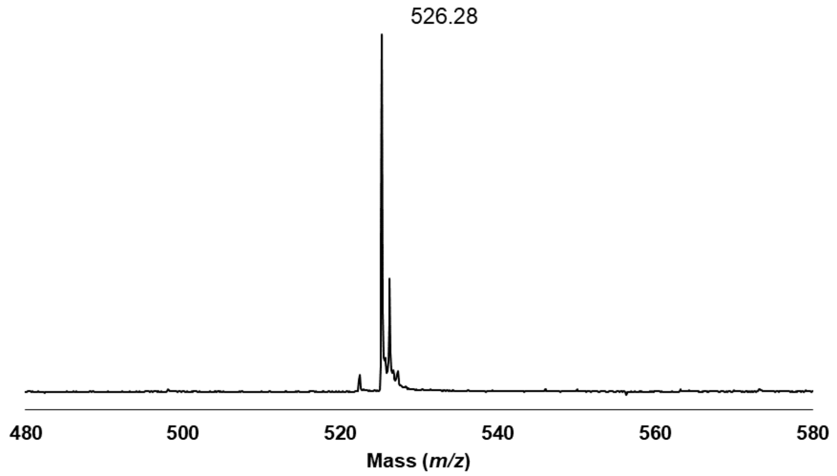

Figure S3. ESI-TOF mass spectrometry of FA-DAP-NH_2. ESI-MS [*m*/*z*]: 526.28 (M + H)+ [Theorical mass: 525.24].

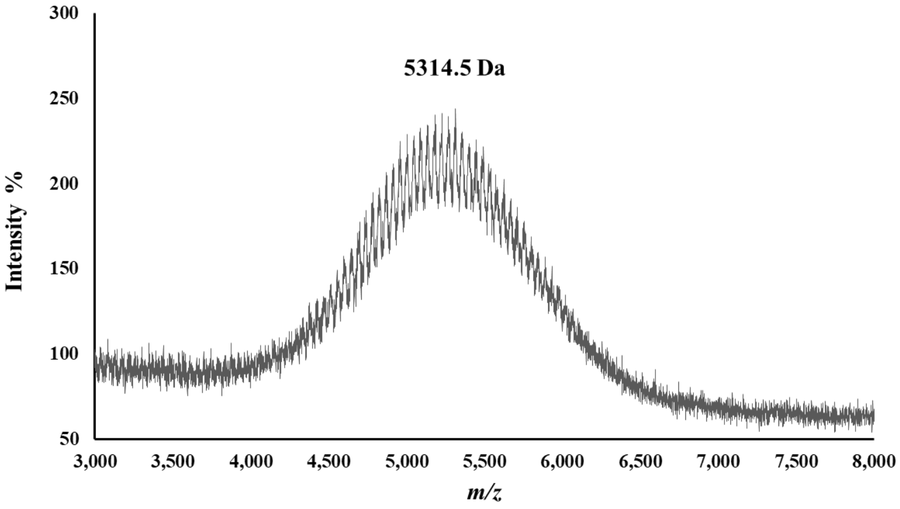

Figure S4. MALDI-TOF of Boc-PEG-FA.

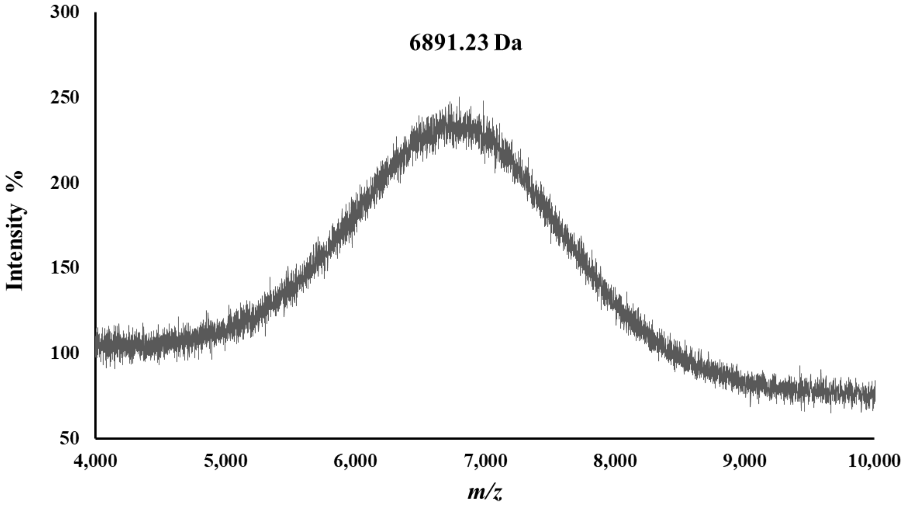

Figure S5. MALDI-TOF of Boc-PEG-$(FA)_3$.

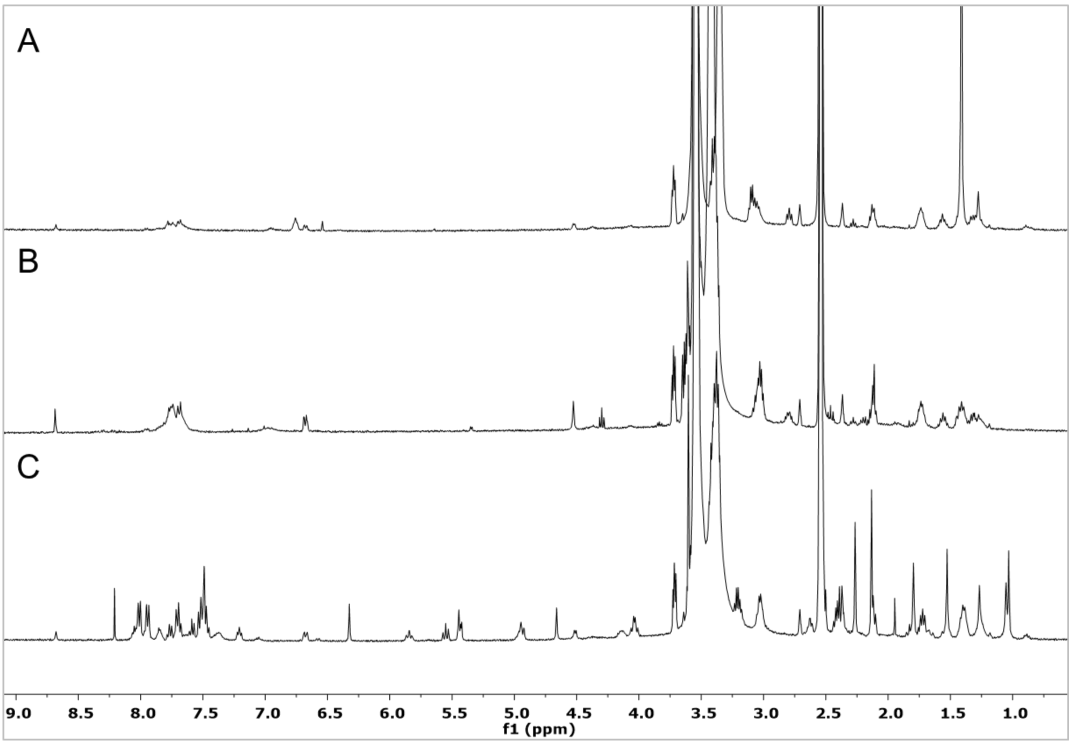

Figure S6. Comparison of ^{1}H-NMR of Boc-PEG-FA (**A**), H_2N-PEG-FA (**B**) and PTX-PEG-FA (**C**) in $(CD_3)_2SO$.

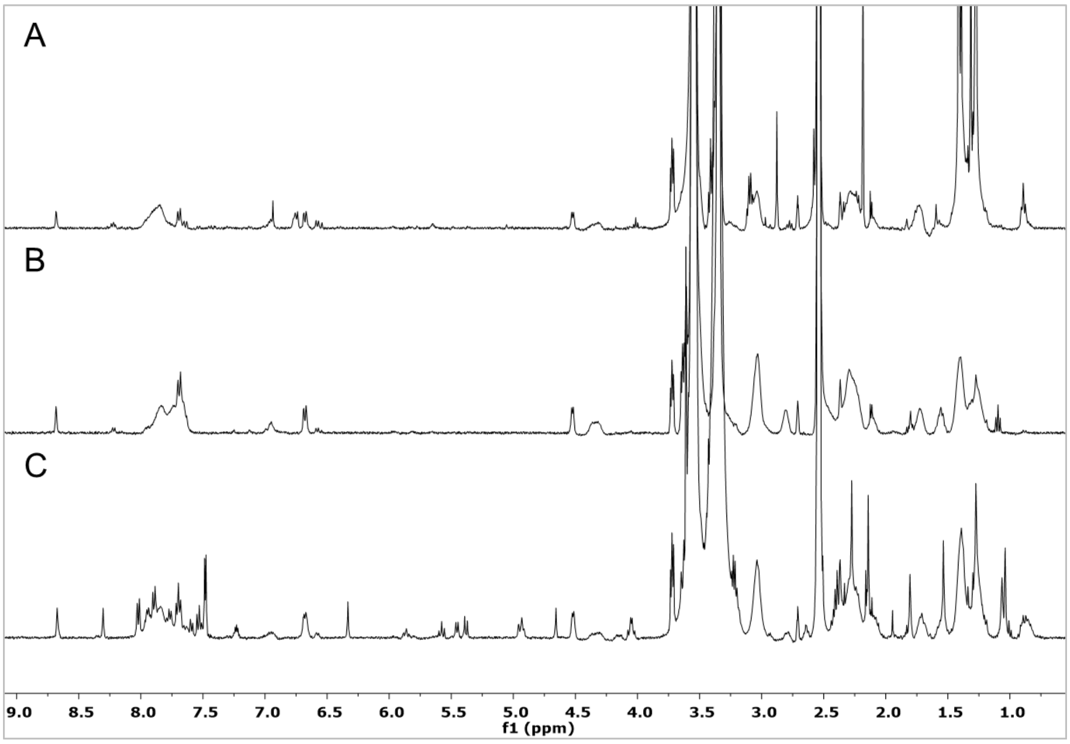

Figure S7. Comparison of ^{1}H-NMR of Boc-NH-PEG-$(FA)_3$ (**A**), H_2N-PEG-$(FA)_3$ (**B**) and PTX-PEG-$(FA)_3$ (**C**) in $(CD_3)_2SO$.

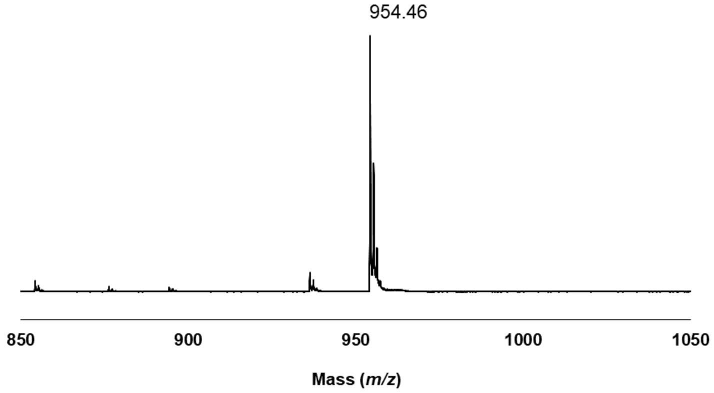

Figure S8. ESI-TOF mass spectrometry of SPTX. ESI-MS [*m/z*]: 954.46 $(M + 1H)^+$ [Theorical mass: 953.35].

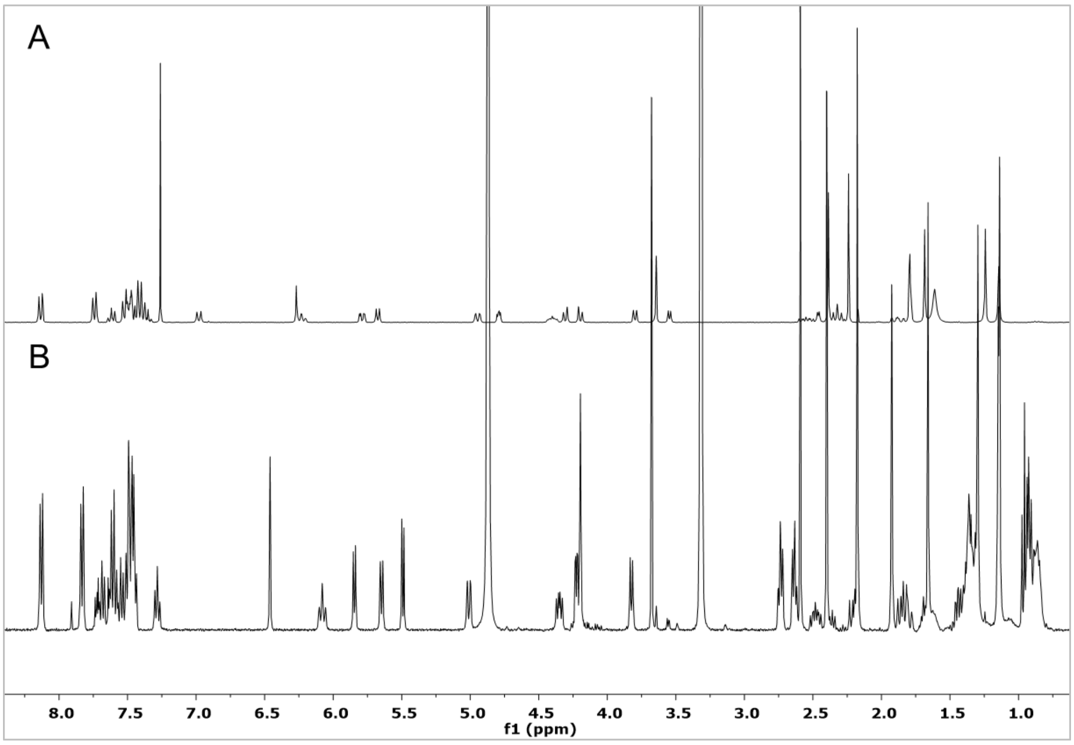

Figure S9. Comparison of ^{1}H-NMR of PTX (**A**) and SPTX (**B**) in CD_3OD.

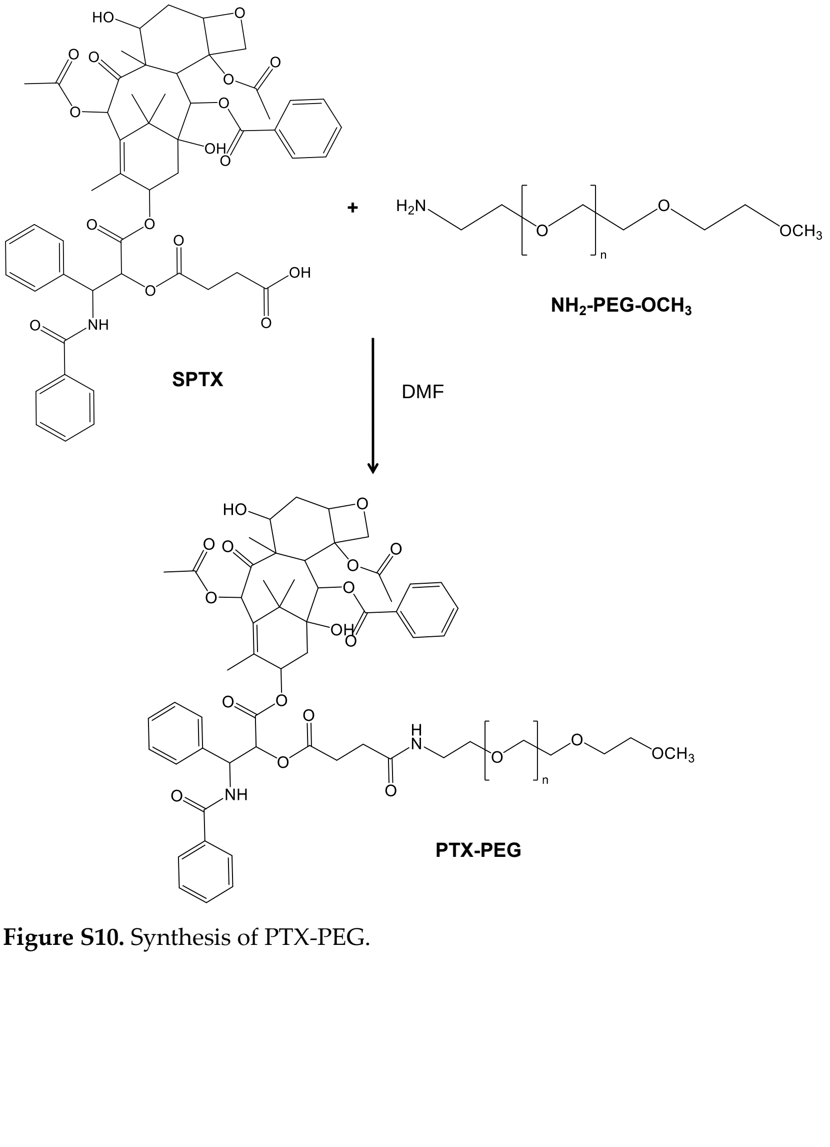

Figure S10. Synthesis of PTX-PEG.

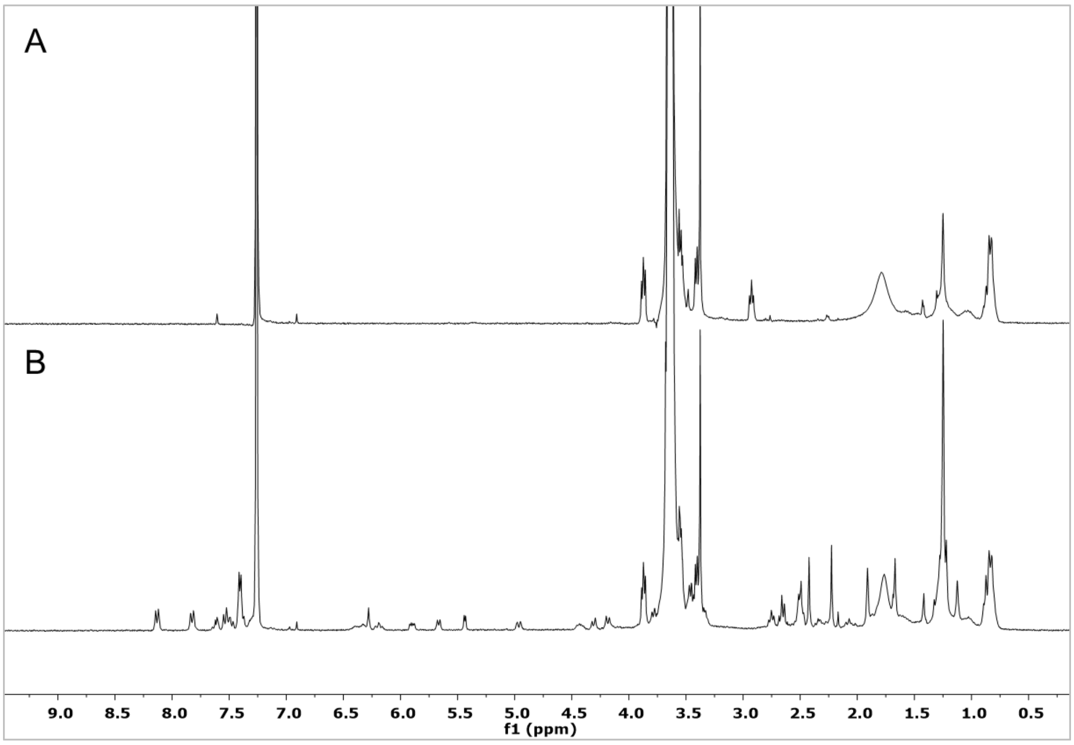

Figure S11. Comparison of ^{1}H-NMR of mPEG-NH_2 (**A**) and PTX-PEG (**B**) in $CDCl_3$.

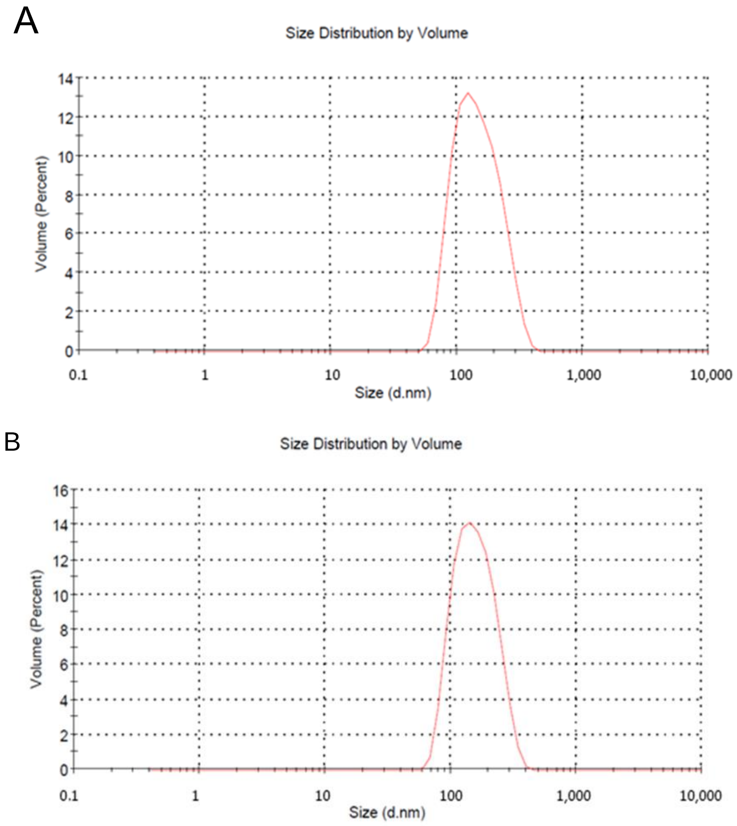

C

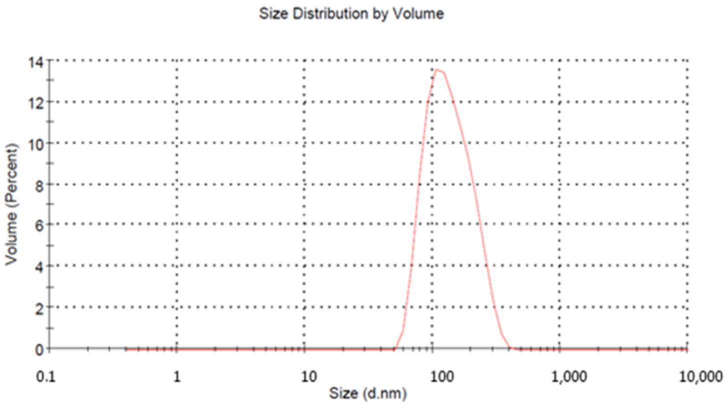

Figure S12. Size distribution by DLS of PTX-PEG (**A**), PTX-PEG-FA (**B**) and PTX-PEG-$(FA)_3$ (**C**).

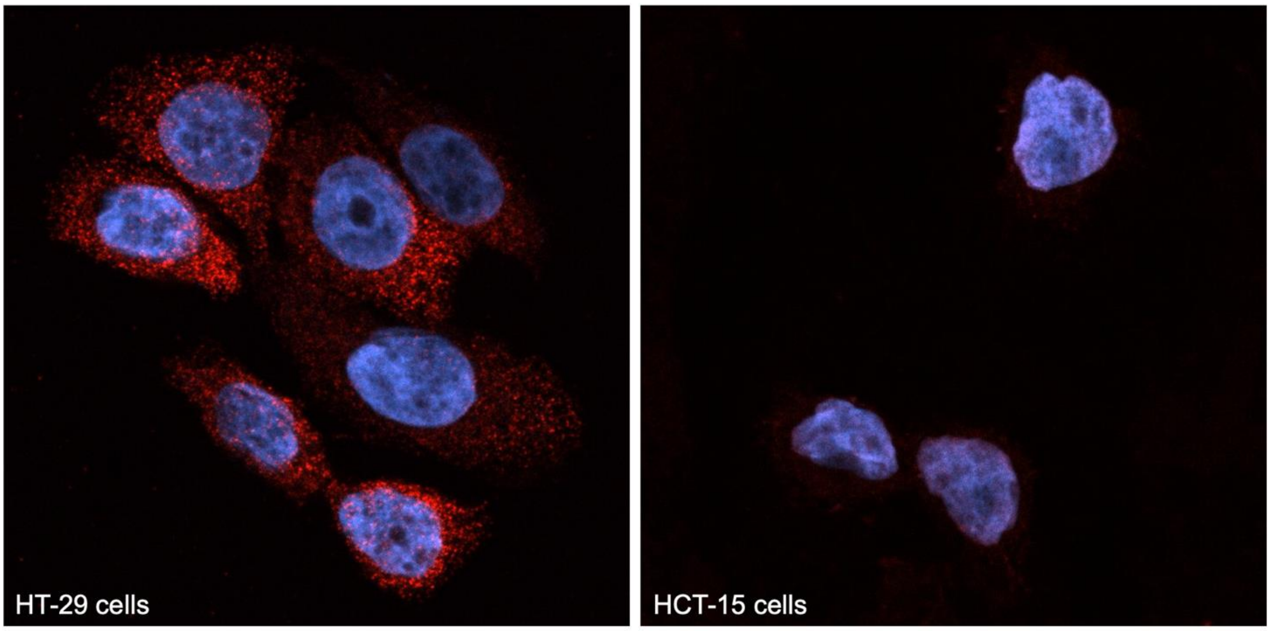

Figure S13. Expression level of FA receptors in HT-29 and HCT-15 cell lines. Magnification: 63×.
